# Transcriptomic and DNA Methylation Profiles of Alternative Aphid Morphs and Genotypes

**DOI:** 10.1002/ece3.73634

**Published:** 2026-05-01

**Authors:** Zhe Yang Yim, Christopher Murgatroyd, Reinmar Hager

**Affiliations:** ^1^ School of Biological Sciences, Faculty of Biology, Medicine and Health University of Manchester Manchester UK; ^2^ Department of Biology University of Oxford Oxford UK; ^3^ School of Healthcare Science Manchester Metropolitan University Manchester UK

**Keywords:** aphid, DNA methylation, polyphenism, transcriptomic, wing‐development

## Abstract

Pea aphids can develop alternative morphs when exposed to stressful conditions, showing differences between genotypes in their stress responses. Although several biological pathways regulating alternative morph production have been investigated, the role of epigenetic mechanisms remains understudied in genotype dependent stress responses. Understanding the underlying differences in stress response between aphid genotypes may provide clues to their adaptation. Here, we use RNA‐seq and MBD‐seq to analyse the transcriptome and DNA methylome profile of alternative morphs in two aphid lineages that differ in their life history and stress response. While most differentially expressed genes between morphs were linked to either wing development or stress responses, these expression differences were not associated with clear DNA methylation profiles. In addition, the transcriptome profile reveals some possible genes in the neuroendocrine pathway and histone modification that could play an important role in regulating the stress response between aphid genotypes. Overall, our study reveals both novel genes and pathways associated with polyphenism in aphids but finds no association between DNA methylation and gene expression.

## Graphical abstract

Graph showing the correlation between differentially expressed genes and differentially methylated genes. There is no correlation between gene expression and DNA methylation level in pea aphid polyphenism.

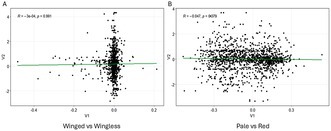

## 1 Introduction

Across insects, many species can produce alternative morphs, such as dispersing winged and non‐dispersing wingless morphs. In most cases, the winged and wingless morphs develop in response to changes in environmental conditions (Hayes et al. 2024). Wing polyphenism clearly acts as an adaptation to stressful conditions as it allows dispersal and escape from unfavourable conditions (Salces‐Castellano et al. 2021). In addition, pea aphids can be found in different body colour morphs, such as red and green. The differences in the body colour of pea aphids enable them to adapt to different environments and may be affected by a wide range of factors such as temperature, endosymbionts, or predation.

Pea aphids are phloem‐sucking insects (Aphidoidea/Hemiptera) and are a serious pest that depresses crop production and also plays an important role as a vector for various plant viruses (Paudel et al. 2018). Pea aphids have a complex life history and reproductive patterns (Reyes et al. 2019) and are comprised of multiple populations referred to as ‘biotypes/genotype’ that started diverging around 500,000 years ago (Fazalova and Nevado 2020). A given genotype may exhibit a range of polymorphisms such as body colour polymorphism, and different pea aphid genotypes are found in many colours such as green, red, and black. Wing polyphenism occurs in many genotypes, whereby a single genotype gives rise to multiple discrete phenotypes in response to the surrounding environment (Simpson et al. 2011). Wing dimorphism in aphids can be triggered by different environmental factors such as an increase in density, decrease in plant quality, increase in temperature, or starvation (Parker et al. 2021; Chen et al. 2019; Richard et al. 2019). Interestingly, the ability of aphids to respond to environmental stress varies depending on the genotype. Some genotypes can produce winged offspring in response to stressful environmental conditions while other genotypes do not produce any winged offspring (Wang et al. 2019).

Although many studies have investigated pea aphid polyphenism, the underlying mechanisms are yet to be comprehensively established (Grantham et al. 2016). In male pea aphids, wing polymorphism is regulated by the Api locus located on the X chromosome, with the male carrying the api wl allele being wingless and those with the apiw allele developing wings (Li et al. 2020). However, in the female pea aphid, genotypes can respond differently to environmental conditions by changing body colour (and increased locomotion) or producing winged offspring, but many aspects of the underlying molecular mechanism regulating this polyphenism remain unresolved. Since all offspring that were produced by the mother are genetically identical, the winged aphid and changes in body colour are likely due to changes in epigenetically driven expression differences.

Epigenetic mechanisms are the processes that can alter gene expression without altering any of the underlying DNA sequences. DNA methylation, histone modification and snRNAs are some of the main epigenetic mechanisms. The most commonly studied epigenetic mechanism is DNA methylation. DNA methylation has been implicated for its critical role in regulating polyphenism in insects. For example, a study by Walsh et al. (2010) in the honeybee revealed female‐specific methylation erasure during development and distinct methylation patterns across tissues (such as brain, ovary and sperm) and across developmental stages, suggesting that methylation might play an important role in caste‐differentiation polyphenism. Pea aphids have a complete set of genes that encode the DNA methylation machinery, which includes DNMT1 and DNMT3 (Walsh et al. 2010; Duncan et al. 2022); therefore, it is possible that DNA methylation might play a role in regulating polyphenism in aphids.

In this project, we performed a transcriptional profile and genome‐wide analysis of aphid DNA methylation. We conducted whole‐body analysis instead of tissue‐specific analysis because the main aims of our study were to assess (a) what genes and associated pathways are differentially expressed between the wild‐type (N116 green and N116 red) and their alternative morphs (N116 winged and N116 wingless; N127 pale vs. N127 red); (b) what the underlying transcriptional and methylation profile differences between the two aphid genotypes and their respective morphs are; and (c) whether methylation profile differences are correlated with differences in gene expression. Therefore, we are careful with the interpretation of the methylation level between morphs and genotypes.

## 2 Methods and Materials

### 2.1 Aphids

Two pea aphid genotypes ( *Acyrthosiphon pisum* ), N116 and N127, were used in all experiments due to their differences in response to environmental stress, and were obtained from Colin Turnbull's group at Imperial College, London. The green pea aphid N116 (origin: near Slough, Berkshire, UK) usually produces winged offspring when exposed to crowded conditions. In contrast, the red pea aphid N127 (origin: near Slough, Berkshire, UK) changes its body colour when exposed to crowded conditions (Wang et al. 2019). Aphids were reared on fava bean (*Vicia fabae*, Brand: Abido, www.lemonsalt.co.uk) plants inside a plastic mesocosm container (containing six individual plants) covered with superfine mesh (0.25 mm × 0.8 mm; Allotment‐garden, UK) in a climate‐controlled chamber at 22°C ± 0.5°C, 35% humidity and a 16 h/8 h light–dark cycle. All aphids were maintained in low density populations to avoid the production of alternative morphs due to crowded conditions and had been maintained in the laboratory for at least 1 year prior to beginning the experiments.

### 2.2 Sample Preparation

Six 3‐weeks old fava bean plants were placed into a mesocosm cage, and alternative morph inducing (crowded) conditions were triggered by placing 50 adult female pea aphids on the plants for 14 days. The plants were watered every 3 days followed by randomisation of the mesocosm position in the chamber to eliminate any possible light regime effect. The mesocosm experiment were carried out once for each genotype N116 and N127. The different pea aphid morph samples used in this study (winged, wingless, red, pale) were collected after 14 days. Pooled aphid samples were used with a total number of four biological replicates (each containing seven pea aphids) for each aphid morph and genotype, that is, *n* = 4 for each morph of N116 winged, N116 wingless, N127 pale and N127 red, respectively, for both RNA and MBD‐sequencing.

### 2.3 Transcriptome Sequencing

Total RNA was submitted to the Genomic Technologies Core Facility (GTCF) at The University of Manchester. Quality and integrity of the RNA samples were assessed using a 2200 TapeStation (Agilent Technologies) and then libraries generated using the TruSeq Stranded mRNA assay (Illumina Inc.) according to the manufacturer's protocol. Briefly, total RNA (0.1–4 ug) was used as input material from which polyadenylated mRNA was purified using poly‐T, oligo‐attached, magnetic beads. The mRNA was then fragmented using divalent cations under elevated temperature and then reverse transcribed into first strand cDNA using random primers. Second strand cDNA was then synthesised using DNA Polymerase I and RNase H. Following a single ‘A’ base addition, adapters were ligated to the cDNA fragments, and the products were then purified and enriched by PCR to create the final cDNA library. Adapter indices were used to multiplex libraries, which were pooled prior to cluster generation using a cBot instrument. The loaded flow‐cell was then paired‐end sequenced (76 + 76 cycles, plus indices) on an Illumina HiSeq4000 instrument. Finally, the output data was demultiplexed (allowing one mismatch) and BCL‐to‐Fastq conversion was performed using Illumina's bcl2fastq software, version 2.20.0.422.

### 2.4 Differential Expression Analysis

Raw reads from an Illumina HiSeq 4000 sequencer were assessed by FastQC (http://www.bioinformatics.babraham.ac.uk/projects/fastqc/). Sequence adapters were removed, and reads were quality trimmed using Trimmomatic_0.39 (Bolger et al. 2014). The reads were mapped against the reference *Acyrthosiphon pisum* genome v3.0 and counts per gene were calculated using Structural Annotation OGS3.0 using STAR_2.7.2b (Dobin et al. 2013). The genome assembly and structural annotation were obtained from the Bioinformatics Platform for Agroecosystem Arthropods (BIPAA). Normalisation, principal components analysis, and differential expression were calculated in DESeq2_1.20.0 using default settings (Love et al. 2014). Blast2GO software (http://www.geneontology.org) was used for gene ontology (GO) annotations. Kyoto Encyclopedia of Genes and Genomes (KEGG) analysis was used to identify significantly enriched metabolic pathways or signal transduction pathways in DEGs based on the database with the criteria of *p*‐adjusted value < 0.1 and log_2_ fold change > 0.3 using ggkegg (Sato et al. 2023). These thresholds were selected to capture modest yet biologically meaningful transcriptional changes. In developmental plasticity systems such as wing polyphenism, phenotypic differences usually involve coordinated regulation of multiple genes, where the expression changes at individual genes might be subtle rather than large.

### 2.5 MBD‐Seq Library Preparation and Sequencing

DNA was extracted from pooled samples of seven aphids from each of a total of seven mesocosms for each biological replicate using the Qiagen DNeasy Blood & Tissue Kit (Qiagen, UK) according to the manufacturer's instructions. DNA concentration and quality check were done using nanodrop, gel electrophoresis and qubit (Thermo Fisher Scientific, USA). Genomic DNA was sheared using Next Gen Bioruptor to a size range for 400 bp and was checked using TapeStation System 4200 (Agilent Technologies, USA). DNA enrichment was performed using the MethylMiner Methylated DNA Enrichment Kit (Thermo Fisher Scientific, USA) with an initial input of 1 ug of sheared DNA per reaction. Briefly, DNA was bound to the MBD‐biotin protein containing Dynabeads M‐280 Streptavidin followed by a series of washing. Lastly, the methylated beads were eluted from the capture beads using a High Elution Buffer. Next, the library was prepared using the Next Gen DNA library kit (Active Motif, USA). The eluted DNA underwent two rounds of repair steps and washing, followed by a ligation step with the addition of a specific index (DNA with unique sequence) for each sample using the Next Gen Indexing Kit (Active Motif, USA) for sample identification after sequencing, followed by washing. Lastly, the sample containing specific index underwent a last round of ligation with washing and amplification using PCR at 98°C for 30 s, followed by 6 cycles of DNA denaturation at 98°C for 10 s, followed by annealing at 60°C for 30 s and lastly extension at 68°C for 60 s. The amplified library was then washed and its size analysed using TapeStation System 4200 (Agilent Technologies, USA). The library was sequenced on the Illumina Next Seq 550 Series (illumina, USA) using the NextSeq 500/550 v2.5 Kits (75 cycle) (Illumina, USA). Finally, the output data was demultiplexed (allowing one mismatch) and BCL‐to‐Fastq conversion was performed using Illumina's bcl2fastq software, version 2.20.0.422.

### 2.6 Differential Methylation Analysis

Raw reads of 76 bp from an Illumina NextSeq550 sequencer were checked using a quality control pipeline consisting of FastQC (http://www.bioinformatics.babraham.ac.uk/projects/fastqc/) and FastQ Screen (https://www.bioinformatics.babraham.ac.uk/projects/fastq_screen/). The reads were trimmed to remove any adapter or poor‐quality sequence using Trimmomatic v0.39. Reads were truncated at a sliding 40 bp windows, starting 5′ with a mean quality of <Q20 and removed if the final length was less than 35 bp. The filtered reads were mapped against the reference *Acyrthosiphon pisum* genome v3.0 using Bowtie2 v2.5.2 (Langmead and Salzberg 2012). MACS2 was used to identify MBD regions, and programs from the UCSC repository (http://hgdownload.soe.ucsc.edu/admin/exe/) were used to convert bedGraph files from MACS2 (version 2.2.5) (Zhang et al. 2008) to bigWig, including bedClip and bedGraphToBigWig using the parameters (‐‐format BAM ‐‐gsize 540000000 ‐‐keep‐dup 1 ‐‐bdg ‐‐SPMR ‐‐qvalue 0.05 ‐‐nolambda ‐‐extsize 350).

Differential binding analysis was performed using DiffBind v3.4.11 (Stark and Brown 2011) in R v4.1.2 (R Core Team 2021). The peak input consisted of the candidate MBD region coordinates, in BED format, output by MACS2 (chr, start, end, *q*‐value). The ‘read’ input were the final filtered BAM files used in the MACS2 peak calling. The annotation of differentially bound regions was performed using ChIPseeker v1.30.3 (Yu et al. 2015) Differentially bound MBD regions were filtered by log2 fold change (positive > 0.3 or negative < −0.3) and were grouped together, or analysed individually into hypermethylated (positive log2fold change) or hypomethylated (negative log2fold change) regions using the mean normalised reads of 6.64 (~100 reads).

The relative location of each differentially bound region to associated genes was determined using the annotatePeak function. Gene Ontology enrichment analysis was performed using the enricher function of clusterProfiler v4.2.2 in R v 4.1.2 (Yu et al. 2012) and GO annotation for aphid genes was obtained from the Bioinformatics Platform for Agroecosystem Arthropods (BIPAA). Some GO terms were provided as Enzyme Commission (EC) terms. The EC2GO terms were translated using vlookup in excel and the resulting file provided the GO term and description of each gene. GO terms were divided into Biological Process (BP), Cellular Component (CC) and Molecular Function (MF) groups. The universe (or background) list was generated from all genes represented in the GFF file. ClusterProfiler was used for MBD‐seq data instead of Blast2GO as it allows for seamless downstream analysis of the genomic regions and also associated gene‐level annotations. In contrast, Blast2GO were used for RNA‐seq to assign GO term to transcript, which helps provide insight into biological process and beneficial specifically for non‐model organisms that lacks comprehensive genome annotation.

To investigate the correlation between DNA methylation and gene expression, we integrated MBD‐seq and RNA‐seq datasets using RNAChipIntegrator. Briefly, MBD‐seq regions (chromosome, start, end) were associated with annotated genes (gene ID, chromosome, start, end, strand) derived from RNA‐seq for both group comparisons (N116 winged vs. N116 wingless and N127 pale vs. N127 red). Differentially expressed genes were represented as log2fold changes (LFC) obtained from RNA‐seq analysis. MBD‐seq regions were annotated to nearby genes by assigning regions within ±10 kb of gene transcription start sites (TSS) or transcription end sites (TES) using RNAChipIntegrator. Genes with Not Available (NA) values in RNA‐seq that were associated with MBD‐seq regions were excluded from the analysis. Spearman's rank correlation analysis was performed to assess the relationship between methylation signal intensity and gene expression log2fold (LFC) at a global level. Statistical significance was determined using *p*‐values, with multiple testing correction applied using the Benjamini‐Hochberg false discovery rate (FDR) method. Next, the Differentially Methylated Region (DMR) (the genomic regions that showed differential methylation levels between the two group comparison) were annotated against genomic features which include promoter, exons, UTRs and intergenic regions using GenomicRanges packages in R. The DMR that overlaps with each feature were counted and the expected count was calculated using the relative sizes of the genomic features. Enrichment of DMRs in specific regions was calculated using Fisher's exact test.

## 3 Results

### 3.1 RNA‐Seq Analysis

Beginning with the RNA‐seq analysis, we profiled the four different morphs from two genotypes: N116 (wingless and winged morph) and N127 (red and pale morph) (Hesketh et al. 2025). First, we analysed the overall variation of the samples in our dataset using principal component analysis (PCA). There is a clear separation of clusters between the wild‐type morphs (N116 wingless and N127 red) and alternative morphs (N116 winged and N127 pale), which revealed that most transcriptome variation is caused by the different morphs (Figure S1A,B), but also that the two different genotypes were clearly separated into two different clusters (Figure S1C).

### 3.2 Mapping

Comparing the transcriptomes of the four different pea aphid morphs, we identified many genes and pathways that may play a key role in wing development, body colour, the trade‐off between reproduction and flight, cuticle synthesis, and metabolism in the pea aphid. For RNA‐seq, over 10 million reads were obtained after removing low quality sequences, adapter sequences and ambiguous nucleotides (Table S1). The clean read was mapped to the pea aphid genome, and the mapping ratio ranges from 35% to 88%. Some of the low mapped samples could be due to endosymbionts as aphids usually harbour obligate and facultative symbionts. However, all samples have sufficient reads to be used for downstream analysis.

### 3.3 Differentially Expressed Genes, GO and KEGG Pathways

For N116, 2308 genes were significantly differentially expressed (*p*‐adjust < 0.1, −0.3 < log2fold > 0.3) between winged and wingless morphs. Of the 2308 genes, we identified 44 genes that are potentially involved in regulating wing development in aphids based on previous research (Table S2) based on their potential role in wing development in other insects. These genes were involved in diverse functions such as formation of wings, lipid metabolism, DNA methylation regulation, hormone regulation and also wing expansion behaviour, for example, *Fl, Mad4*, and *pburs*, which have shown to be essential in wing development and expansion behaviour in drosophila. 2308 genes with (*p*‐adjust < 0.1 and −0.3 < log2fold > 0.3) from the DEGS gene list were subjected to GO and KEGG analysis (Figures S2 and S3).

In N127, 2076 genes were significantly differentially expressed (*p*‐adjust < 0.1, −0.3 < log2fold > 0.3) between the red and pale morph. Of these, we identified 40 genes based on their potential role in regulating aphid body colour morphs and development. These genes were involved in carotene production, reproduction, metabolism, longevity, and cuticle formation (Table S3), and GO and KEGG analyses showed these were enriched in 11 and 16 pathways, respectively (Figures S4 and S5).

Next, we were interested in the transcriptomic differences between the two genotypes. Here, 5108 genes were significantly differentially expressed (*p*‐adjust < 0.1, −0.3 < log2fold > 0.3) between the N127 red aphid and N116 green wingless aphid, that is, the normal, un‐stressed morphs of either genotype. Of the 5108 genes, 50 genes were identified that may play a role in regulating their response to stress. For example, many genes were involved in histone modification, neuroendocrinal signalling (Table S4). Since neuroendocrinal signalling such as juvenile, ecdysone and the insulin pathways have been shown to regulate wing development in insects, it is possible that these genes are responsible for the different stress response (producing winged morphs or changing body colour). GO and KEGG analysis for 1737 genes with (*p*‐adjust < 0.1 and log2fold > 1) revealed enrichment for 11 GO terms and 16 KEGG pathways (Figures S6 and S7).

### 3.4 Methylome Profiles of Different Pea Aphid Morphs and Genotype

We next profiled all four morphs for their DNA methylome. Across all samples, MBD‐seq yielded over 35 million reads (after removing low quality reads, adapter sequences; Table S5). The clean reads were mapped to the aphid genome at the mapping ratio average of 74%. First, we analysed the overall variation of the samples in our dataset using principal component analysis (PCA). There was a clear separation of clusters between the wild‐type morphs (N116 wingless and N127 red) and alternative morphs (N116 winged and N127 pale), which revealed that most methylome profile variation is caused by the different morphs (Figure S8A,B). However, the two different genotypes were also clearly separated into two different clusters (Figure S8C).

### 3.5 Genome Wide Methylation Differences Between Aphid Morphs and Genotypes

**FIGURE 1:**
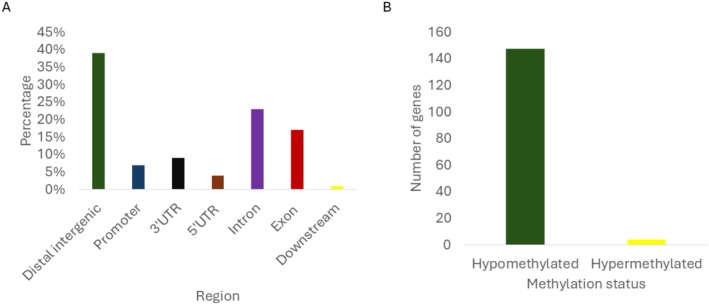
MBD‐seq results for N116 winged and N116 wingless aphids. (A) The DMR overlap region between N116 winged and wingless. Dark blue = promoter, orange = 3′UTR, grey = 5′UTR, yellow = intron, light blue = exon, green = distal intergenic, black = downstream. (B) The total number of genes that were differentially methylated (genes that overlapped with DMRs). Green = hypermethylated, yellow = hypomethylated.

In N116, most DMRs are located within the gene body (exon + intron; 39.5%) followed by the distal intergenic (38%), UTR (13.5%), and promoter regions (7%) (Figure 1A). However, once we consider the relative size of each location, we found that the gene body region was not significantly enriched in comparison to other regions (*p* < 0.001, Odds Ratio = 0). 147 genes were found to be hypomethylated (decreased methylation in N116 winged compared to N116 wingless), while four genes were hypermethylated (increased methylation in N116 winged compared to N116 wingless; Figure 1B).

The top 10 most hypomethylated genes were mostly involved in ubiquitin processes while all four most hypermethylated genes were involved in cellular development (Table S6). No significant enrichment was found for any of the genes.

**FIGURE 2:**
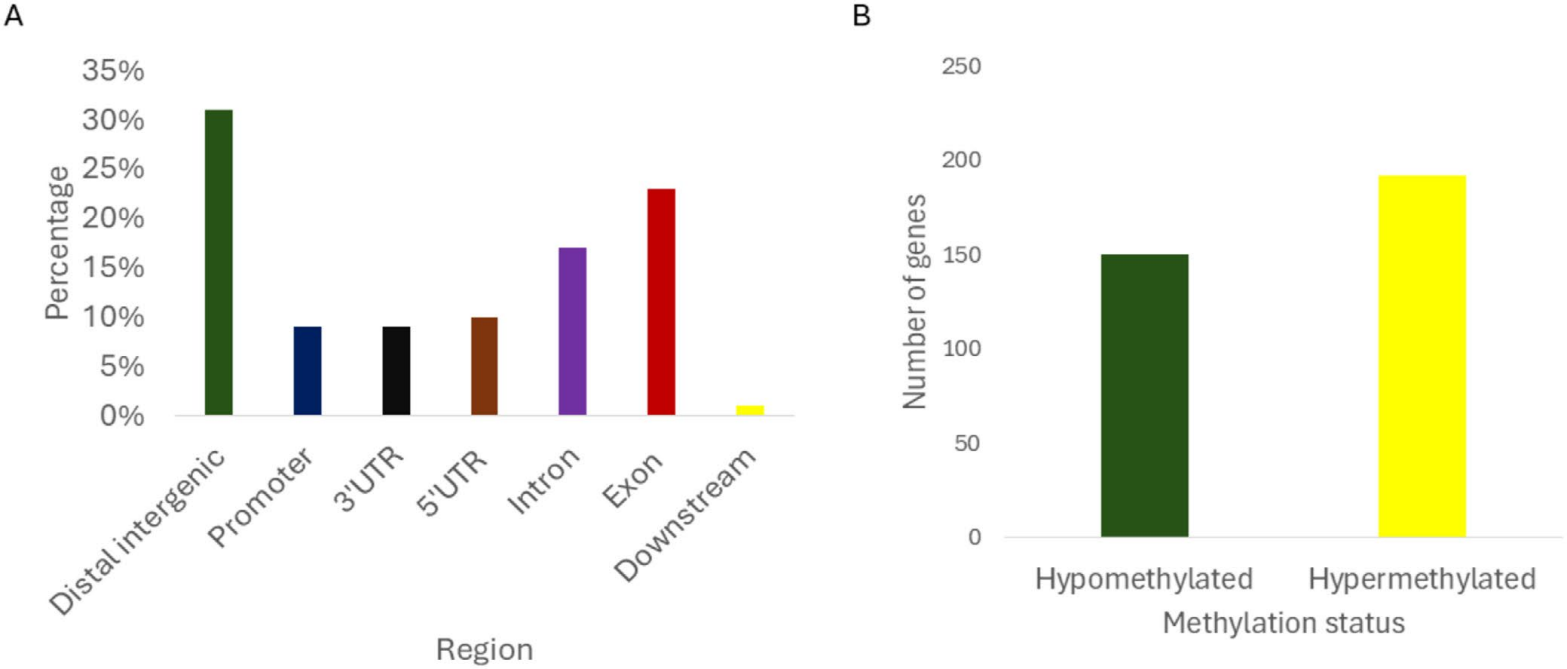
MBD‐seq results for N127 pale and N127 red aphids. (A) The DMR overlap region between N127 pale aphid vs. N127 red aphid. Dark blue = promoter, orange = 3′UTR, grey = 5′UTR, yellow = intron, light blue = exon, green = distal intergenic, black = downstream. (B) The total number of genes that are differentially methylated (genes that overlapped with DMRs). Green = hypermethylated, yellow = hypomethylated.

In N127, the most DMR falls under gene body (exon + intron) (40%), followed by distal intergenic (31%), UTR (19%), promoter (9%), and downstream (1%) regions (Figure 2A). Again, once we consider the relative size of each genome location, the gene body region was not significantly enriched in comparison to other regions (*p* < 0.001, odd ratio = 0.3). 153 genes were hypomethylated (decreased expression in N127 pale compared to N127 red), and 192 genes were hypermethylated (increased methylation in N127 pale compared to N127 red; Figure 2B).

For N127 the top 10 most hypomethylated genes were mostly involved in cellular development, while the top 10 most hypermethylated genes were involved in signalling, cellular development and histone processes (Table S7). Again, none of the genes showed significant enrichment for either GO or KEGG analysis.

**FIGURE 3:**
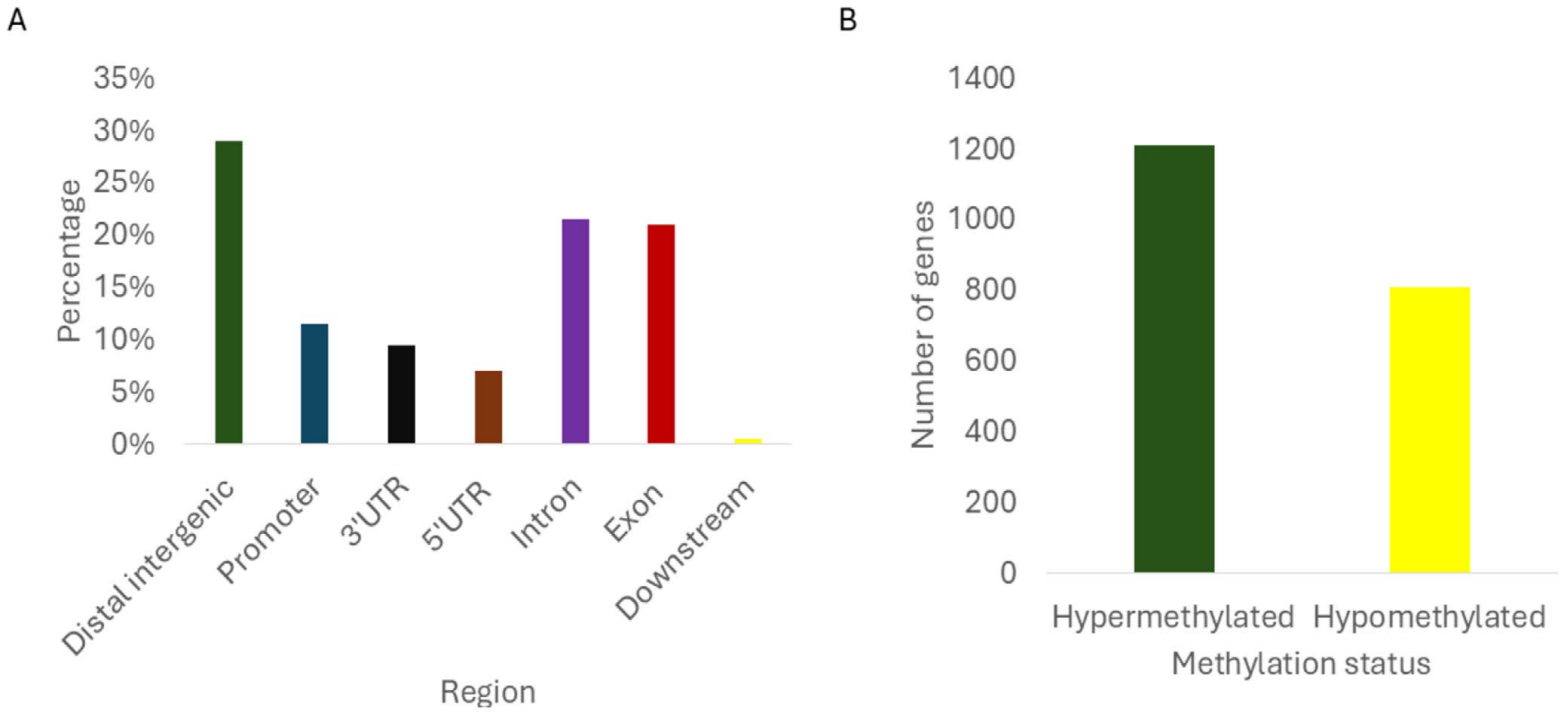
MBD‐seq results for N127 red and N116 wingless aphids. (A) The DMR overlap region between N127 pale vs. N127 red aphids. Dark blue = promoter, orange = 3′UTR, grey = 5′UTR, yellow = intron, light blue = exon, green = distal intergenic, black = downstream. (B) The total number of genes that are differentially methylated (genes that overlapped with DMRs). Green = hypermethylated, yellow = hypomethylated.

Next, we were interested in the methylome profile between the two genotypes (N127 red vs. N116 wingless). Between the two genotypes' unstressed morphs (N127 red vs. N116 wingless), most DMR fall under gene body (exon + intron) (42.5%), followed by distal intergenic (29%), UTR (16.5%), promoter (11.5%) and downstream (0.5%) regions (Figure 3A). 807 genes were hypomethylated (decreased methylation in N127 red comparison to N116 wingless) and 1210 genes were hypermethylated (increased methylation in N127 red compared to N116 red; Figure 3B). Most of the top 10 hypomethylated and hypermethylated genes have uncharacterised function with some involving in cuticle formation and immunity (Table S8). All 2017 differentially methylated genes with (*p*‐adjust < 0.1 and log2fold > 1) list were examined in a GO analysis and showed enrichment in three enriched GO terms (Figure S9).

### 3.6 Correlation of DNA Methylation and Gene Expression

**FIGURE 4:**
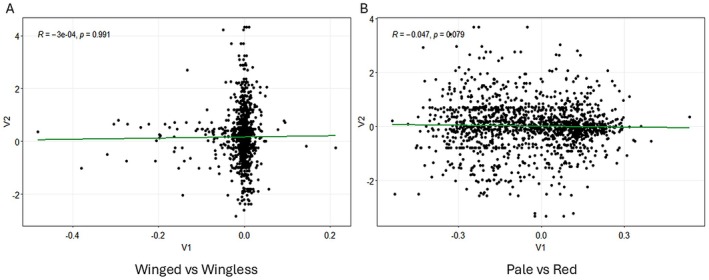
Correlation between differentially methylated regions from MBD‐seq with genes from RNA‐seq at a global level. (A) N116 winged vs. N116 wingless. X‐axis represents the log2fold change of the DMR sites and the y‐axis represents the log2fold change of gene expression on RNA‐seq that overlap with MBD‐seq. (B) N127 pale vs. N127 red. X‐axis represents the log2fold change of the DMR sites and the y‐axis represents the log2fold change of gene expression on RNA‐seq that overlap with MBD‐seq.

To provide initial insight into the relationship between DNA methylation and gene expression, we first examined whether differentially methylated regions found in the MBD‐seq overlapped with genes found in the RNA‐seq. Our result showed, at a global level, DNA methylation was not associated with gene expression across both group comparisons (Rho = −0.0003, *p* = 0.991, Rho = −0.047, *p* = 0.079) (Figure 4).

## 4 Discussion

Our main aim was to determine the transcriptomic and methylome profile of different pea aphid polyphenic morphs (winged vs. wingless and red vs. pale). We further aimed to begin establishing the underlying transcriptional and methylome profile of two different pea aphid genotypes that respond very differently to environmental stressors, namely producing winged offspring and changing body colour morphs. Our expression data showed that genes involved in wing formation were differentially expressed between the N116 winged and N116 wingless morphs. In contrast, metabolic and carotenoid genes were differentially expressed between the N127 red and N127 pale morphs. Our data also found that genes involved in neuroendocrinal signalling and histone modification were differently expressed between the two genotypes. Our methylation results also reveal that the gene body is highly differentially methylated between morphs and genotypes, consistent with other studies that found similar results in different insect species (Hesketh et al. 2025; Xu et al. 2021). Lastly, our data reveal that gene expression is not correlated with methylation between aphid morphs.

### 4.1 Transcriptional Profiles of Aphid Morphs

Wing development genes such as *fl, TnC, Mad* and *Pburs* have been shown to be essential for wing development in drosophila and other insects (Lu et al. 2023; Sun et al. 2025; Zecca and Struhl 2021; Li et al. 2021). We found that these genes were also highly expressed in the winged morphs in aphids and therefore might be essential for regulating wing development in the pea aphid. In addition, chemosensory genes such as *ORF2*, *ORF4* were also more highly expressed in the winged morphs. During dispersal, winged morphs rely on volatile chemical cues to locate new host plants. As a result, they may require a more developed chemosensory system, which allows them to perceive these cues more accurately. Our findings align well with previous studies that reported a similar increase in chemosensory gene expression in the winged morphs of cotton aphids (*Aphid gossypii*) (Peng et al. 2021). We also found a higher level of expression of apolipoprotein D (*ApoD*) and the G‐protein coupled receptor *Mth2 i*n the winged morph. These genes are associated with increased expression during starvation conditions in insects (Zhao et al. 2025; Qiao et al. 2025) which might help explain increased lifespans and starvation resistance in the winged aphid. In addition, many genes involved in histone modification, such as histone‐lysine N‐methyltransferase E(z), histone acetyltransferase p300 (*EP300*), were found to be downregulated in the winged morphs (Tong et al. 2024; An et al. 2025), suggesting that histone modification might be another important epigenetic mechanism in regulating aphid polyphenism.

In contrast, the N127 genotype showed no differential expression in wing related genes. Instead, most genes that were differentially expressed were related to metabolism, insulin signalling, and carotene synthesis/breakdown pathways. For example, the insulin receptor substrate 1 (*IRS*), insulin‐like peptide receptor (*ILPR*), apolipoprotein D (*ApoD*), and forkhead protein O (*FoxO*) were all upregulated in the pale morphs. Similar observations were made in other insects during starvation which suggests that these genes are crucial for survival under starvation conditions (Qiao et al. 2025; Li et al. 2024). Furthermore, genes involved in carotene biosynthesis, including phytoene desaturase (*PDS*) and phytoene synthase (*PSY*), were also differentially expressed between the pale and red morphs, which could potentially contribute to the body colour differences observed between the two morphs (Ding et al. 2022).

We note, however, given the critical period of wing development in aphids starts from the 3rd instar, the changes in gene expression (and indeed methylation) might not capture the dynamics of gene expression differences occurring throughout wing development. This is further compounded by telescoping generations whereby the adult female may also contain embryos, which may also affect expression and methylation level analyses. Unless the age is standardised, this is a common limitation in this type of experiment.

### 4.2 Transcriptional Profiles of Aphid Genotypes Showing Differential Stress Responses

The two aphid genotypes respond very differently to stress (producing wings or changing body colours). Our transcriptional data reveals a high number of histone‐related genes, such as histone deacetylase complex subunit SAP18 (*Bin1*) and set1/Ash2 histone methyltransferase complex subunit ASH2 (*ash2*), histone acetyltransferase KAT7‐like (*kat7), and* histone deacetylase 8 (*HDAC8*) that were differentially expressed between these two genotypes. These histone modification genes have been shown to regulate development and plastic traits in insects (Kirfel et al. 2020; Choppin et al. 2021). Therefore, it is possible that these underlying histone modifications play an essential role in regulating the stress response between aphid genotypes. Furthermore, a large number of genes involved in the neuroendocrine signalling, such as juvenile hormone acid O‐methyltransferase (*JHMAT*), juvenile hormone epoxide hydrolase 1 (JHEH1), insulin‐like peptide receptor (*ILPR*), ecdysone‐induced protein 78C (*E78*), forkhead box protein O (*FoxO*), and ecdysone receptor (*EcR*), were differentially expressed between the two genotypes. These neuroendocrine genes have been previously shown to be involved in regulating different polyphenisms in a wide range of species (Vellichirammal et al. 2017; Zera 2006; Xu et al. 2015; Yuan et al. 2023). It is possible that these pathways might potentially be involved in the stress response between different aphid genotypes. However, further research involving functional perturbation such as RNAi might be needed to validate the role of these genes in aphid.

### 4.3 Methylation Profiles of Aphid Morphs

Across all group comparisons, most of the DMRs are in the gene body (exon + intron). However, when we consider the relative size of the genomic location, the gene body was not significantly enriched in comparison to other regions. Our results are in concordance with other studies that showed that gene bodies are the most methylated regions across different insect species, such as honeybees, Florida carpenter ants, and Nevada termites (Wang et al. 2020; Glastad et al. 2016; Song et al. 2017). Our methylation data further showed that most of the differentially methylated genes were involved in cellular and translational processes. Our DMR results also did not detect any genes that could potentially be responsible for regulating polyphenism in aphids. A possible explanation is that genes usually involved in insect polyphenism are lowly methylated in comparison to housekeeping or ubiquitously expressed genes. Our results are in accordance with other studies that found low levels of methylation in genes responsible for developmental plasticity and high levels of methylation in genes that are more ubiquitously expressed. Another plausible explanation for lower methylation in genes responsible for plasticity is that it allows these to maintain greater flexibility in their expression. A study in the seagrass *Cymodocea nodosa* showed that genes that usually have lower methylation have higher flexibility in gene expression when exposed to stress conditions (Entrambasaguas et al. 2021). In addition, another study has reported higher chromatin accessibility in low methylated genes (Bogan et al. 2023). Therefore, low methylation might help increase chromatin accessibility for genes involved in polyphenism to allow for a wider range of expression and flexible plasticity during stressful conditions.

Overall, our results provide insights into possible candidate genes involved in the regulation of polyphenism between aphid morphs and also genotypes. However, our results also indicate that DNA methylation does not correlate with gene expression in regulating polyphenism in pea aphids, which is in concordance with other studies that found no correlation between gene expression and DNA methylation. Future work focusing on other epigenetic mechanisms, such as lncRNAs and histone modifications and their role in the wing polyphenism of pea aphids, differences in body colour morphs, and sensing stressors is needed to help provide more insights into the importance of epigenetic mechanisms in aphid wing development.

## Author Contributions

**Zhe Yang Yim:** conceptualization (lead), formal analysis (lead), methodology (lead), validation (lead), visualization (lead), writing – original draft (lead), writing – review and editing (lead). **Christopher Murgatroyd:** methodology (equal), writing – review and editing (equal). **Reinmar Hager:** methodology (supporting), resources (supporting), supervision (lead), writing – original draft (supporting), writing – review and editing (supporting).

## Funding

The authors have nothing to report.

## Ethics Statement

The authors have nothing to report.

## Consent

The authors have nothing to report.

## Conflicts of Interest

The authors declare no conflicts of interest.

## Supporting information


**Data S1:** ece373634‐sup‐0001‐Supinfo.docx.

## Data Availability

The datasets generated and/or analysed during the current study are available in the Annotare2.0/Biostudies repository, https://www.ebi.ac.uk/biostudies/arrayexpress/studies/E‐MTAB‐15213?key=93be0637‐6311‐4305‐bec6‐85505acbffd3 with accession number E‐MTAB‐15213 for RNA‐seq and https://www.ebi.ac.uk/biostudies/arrayexpress/studies/E‐MTAB‐15212?key=17ab5fbb‐e2c0‐480e‐9d71‐b0b60129f162 with accession number E‐MTAB‐15212 for MBD‐seq.
