# Supplementary material for: Transcriptomic and DNA Methylation Profiles of Alternative Aphid Morphs and Genotypes
Source: Ecol Evol. 2026 May 1;16(5):e73634. doi: 10.1002/ece3.73634 (PMC13134927; doi:10.1002/ece3.73634)
Supplement: Supplementary file 1 — Data S1: ece373634‐sup‐0001‐Supinfo.docx. [file ECE3-16-e73634-s001.docx]

**Supplementary data**

**Table 1. Number of reads for each samples for RNA-seq**.

| Strain | sample | Number of input reads | | Uniquely mapped reads number |
| --- | --- | --- | --- | --- |
| N116_winged | SY1 | 12,851,782 | 6,298,196 | |
| N116_winged | SY2 | 14,591,774 | 8,911,283 | |
| N116_winged | SY3 | 25,701,576 | 9,022,842 | |
| N116_winged | SY4 | 25,466,197 | 13,553,598 | |
| N116_wingless | SY5 | 12,448,199 | 9,348,786 | |
| N116_wingless | SY6 | 25,387,620 | 16,972,309 | |
| N116_wingless | SY7 | 27,573,262 | 17,295,996 | |
| N116_wingless | SY8 | 14,874,675 | 9,073,252 | |
| N127_pale | SY9 | 10,212,161 | 8,456,725 | |
| N127_pale | SY10 | 40,217,034 | 33,337,704 | |
| N127_pale | SY11 | 26,931,353 | 22,221,046 | |
| N127_pale | SY12 | 19,533,203 | 15,941,794 | |
| N127_red | SY13 | 19,218,541 | 15,628,644 | |
| N127_red | SY14 | 18,144,682 | 14,799,461 | |
| N127_red | SY15 | 21,062,554 | 17,361,696 | |
| N127_red | SY16 | 23,863,412 | 19,898,119 | |

**Table 2. A subset of differentially expressed genes between N116 winged and N116 wingless aphids from the full list that may be important for wing development in pea aphid.**

| **Gene ID** | **Protein Information from NCBI** | **log2fold** | **p-value adjusted** |
| --- | --- | --- | --- |
| LOC100168346 | troponin C | 3.25 | 7.75E-06 |
| LOC100160700 | phosphoenolpyruvate carboxykinase | 1.60 | 1.87E-05 |
| LOC100161369 | flightin | 5.28 | 0.0001 |
| LOC100570184 | nose resistant to fluoxetine protein 6 isoform X1 | 1.13 | 0.0002 |
| LOC100161202 | lipase 3 | 0.51 | 0.0018 |
| LOC100162614 | mothers against decapentaplegic homolog 4 | 0.49 | 0.0073 |
| LOC100159349 | G-protein coupled receptor Mth2 | 0.43 | 0.0081 |
| LOC100165833 | ecdysone 20-monooxygenase isoform X2 | 0.41 | 0.0155 |
| LOC100166489 | eclosion hormone-like | 0.93 | 0.0014 |
| Awd2 | abnormal wing discs 2 isoform X1 | 0.37 | 0.0014 |
| LOC100158736 | facilitated trehalose transporter Tret1 isoform X1 | 1.04 | 0.0191 |
| LOC100169493 | mitogen-activated protein kinase p38b isoform X1 | 0.42 | 0.0191 |
| LOC100166282 | partner of bursicon | 0.67 | 0.0222 |
| LOC100161983 | apolipoprotein D isoform X1 | 0.46 | 0.0279 |
| ORF2 | chemosensory protein-like precursor | 0.54 | 0.0293 |
| ORF4 | chemosensory protein-like | 0.65 | 0.0461 |
| LOC103309827 | DNA methyltransferase 1-associated protein 1 | 0.42 | 0.0776 |
| LOC100168129 | histone-lysine N-methyltransferase, H3 lysine-79 specific | 0.59 | 0.0207 |
| LOC100164834 | Krueppel homolog 1 isoform X3 | 0.69 | 0.0303 |
| LOC100570344 | esterase E4 | -2.11 | 0.0003 |
| LOC100160034 | phenoloxidase 1 | -1.18 | 0.0008 |
| LOC100163455 | heat shock 70 kDa protein 4 | -0.47 | 0.0018 |
| LOC100159933 | isocitrate dehydrogenase | -0.35 | 0.0027 |
| LOC100160397 | heat shock protein 83 | -0.90 | 0.0034 |
| LOC100158703 | optomotor-blind protein | -0.44 | 0.0058 |
| LOC100168026 | heat shock protein 70 B2 | -0.74 | 0.0068 |
| LOC100159778 | glycogen phosphorylase isoform X1 | -0.59 | 0.0085 |
| LOC100165557 | trithorax group protein osa isoform X4 | -0.50 | 0.0288 |
| LOC100159050 | phytoene desaturase | -1.15 | 0.0135 |
| LOC100160060 | protein gustavus isoform X4 | -0.36 | 0.0148 |
| LOC100158806 | protein held out wings isoform X1 | -0.53 | 0.0149 |
| LOC100162796 | homeotic protein spalt-major isoform X2 | -0.63 | 0.0166 |
| LOC100167672 | protein dachsous | -0.79 | 0.0184 |
| LOC100164739 | acetyl-CoA carboxylase | -0.56 | 0.0210 |
| LOC100162023 | histone deacetylase Rpd3 | -0.40 | 0.0320 |
| LOC100574903 | histone-lysine N-methyltransferase eggless | -0.41 | 0.0337 |
| LOC100167980 | homeobox protein homothorax isoform X2 | -0.50 | 0.0342 |
| Atpcl | ATP citrate lyase | -0.34 | 0.0413 |


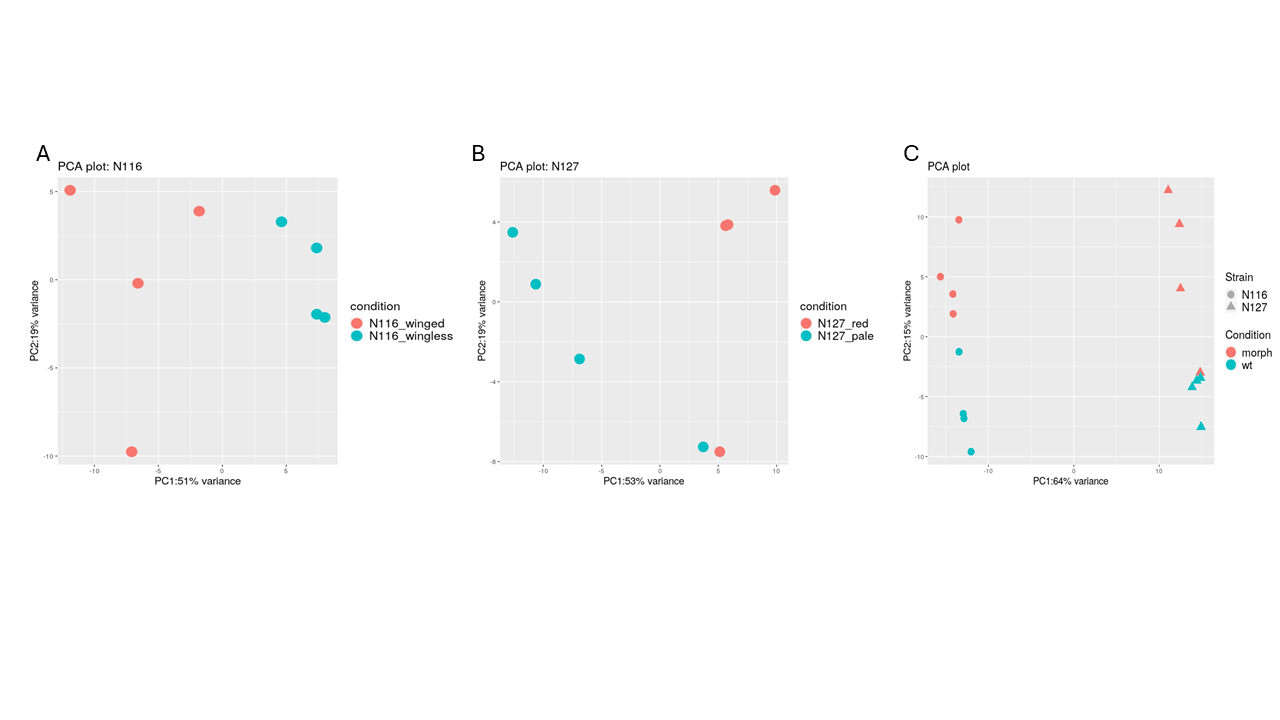


**Figure 1. PCA plot of transcriptome profiles of different aphid morphs.** A) N116 winged vs N116 wingless. Principal component analysis (PCA) shows clustering of RNA-seq samples by morphs. PC1 explains 51% of the total variance, separating the N116 winged morphs from N116 wingless (wild type). PC2, explaining 19% of the total variance, differentiates N116 winged from N116 wingless. B) N127 pale vs N127 red. PC1 explains 53% of the total variance and separates the N127 pale morph from N127 red (wild type). PC2, explaining 19% of the total variance, captures variation such as individual differences within morphs, or environmental effects between N127 pale vs N127 red. C) Full PCA plot with wild type and morphs, the two aphid genotype cluster into two groups.


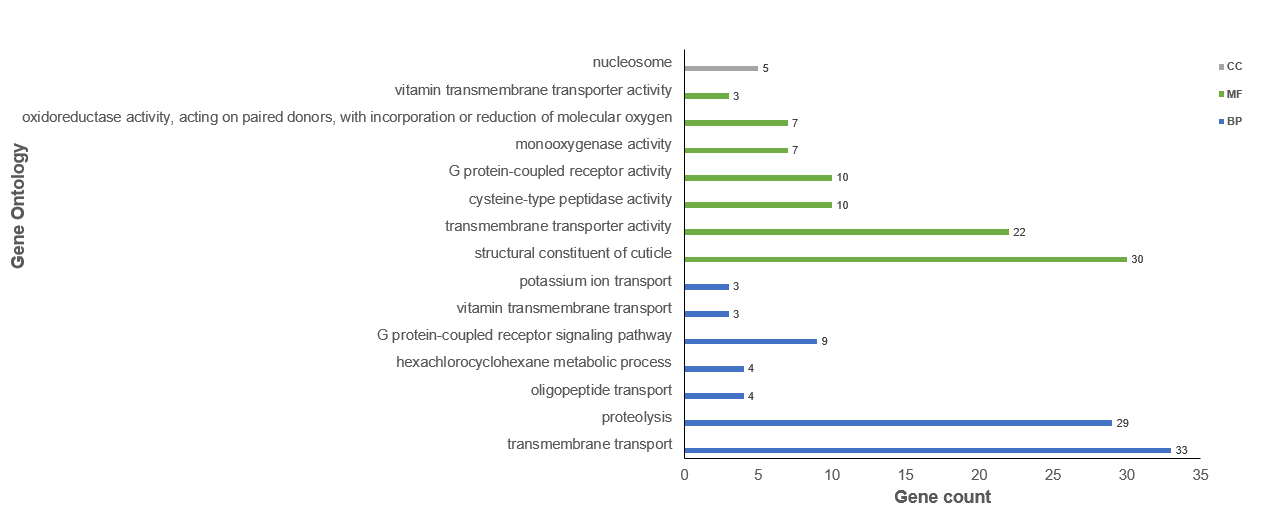
 **Figure 2. Gene Ontology analysis for N116 winged vs N116 wingless.** The colours of the bars represent the intensity of the different GO term. Values represent the number of genes count enriched for each term.


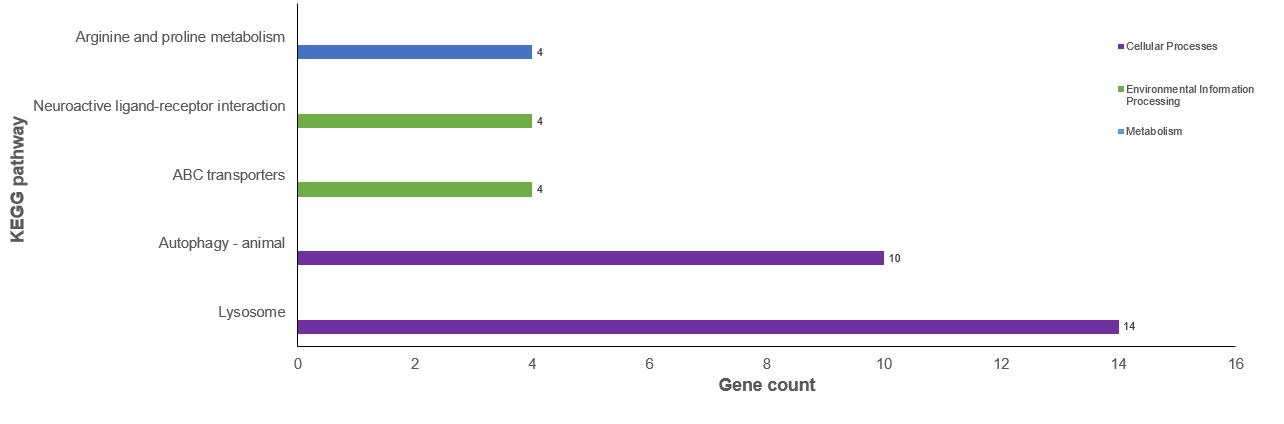


**Figure 3. KEGG analysis for N116 winged vs N116 wingless.** The colours of the bars represent the KEGG pathway enrichment and the values represents the number of genes that were enriched for the kegg pathway.

**Table 3. A subset of differentially expressed genes between N127 pale and N127 red aphids from the full list that could potentially be important for body colour morphs and stress response in aphid.**

| Gene ID | Protein Information from NCBI | log2fold | p-value adjusted |
| --- | --- | --- | --- |
| LOC100167084 | lipid storage droplets surface-binding protein 1 isoform X2 | 1.69 | 1.22E-15 |
| LOC100169576 | facilitated trehalose transporter Tret1 isoform X1 | 3.43 | 1.07E-08 |
| LOC100161594 | short-chain specific acyl-CoA dehydrogenase, mitochondrial | 1.09 | 4.19E-07 |
| LOC100159065 | heat shock 70 kDa protein cognate 4 | 0.57 | 8.94E-07 |
| LOC100165228 | Galactokinase | 1.60 | 2.41E-06 |
| LOC100569479 | insulin receptor substrate 1 isoform X1 | 0.76 | 4.97E-06 |
| LOC100159349 | G-protein coupled receptor Mth2 | 0.68 | 7.67E-06 |
| LOC100160293 | Phosphoglucomutase | 0.86 | 2.84E-05 |
| LOC100169464 | insulin-like peptide receptor isoform X1 | 0.39 | 9.26E-05 |
| LOC100168129 | histone-lysine N-methyltransferase, H3 lysine-79 specific | 0.75 | 0.0003 |
| Usp | ultraspiracle isoform X2 | 0.34 | 0.0068 |
| LOC100168097 | forkhead box protein O | 0.40 | 0.0067 |
| LOC100161983 | apolipoprotein D isoform X1 | 0.43 | 0.0016 |
| LOC100160300 | juvenile hormone epoxide hydrolase 1 | 0.50 | 0.0001 |
| LOC100165833 | ecdysone 20-monooxygenase isoform X2 | 0.53 | 0.0003 |
| LOC103309827 | DNA methyltransferase 1-associated protein 1 | 0.34 | 0.0099 |
| LOC100574398 | trehalase-like | 1.10 | 0.0136 |
| LOC100166514 | octopamine receptor | 0.88 | 0.0205 |
| LOC100168659 | insulin-like receptor | 0.36 | 0.0214 |
| LOC100161832 | Insulin | 0.51 | 0.0415 |
| LOC100161380 | phytoene desaturase | 0.53 | 0.0464 |
| LOC100167145 | heat shock protein 68-like | 0.64 | 0.0481 |
| LOC100160300 | juvenile hormone epoxide hydrolase 1 | 0.50 | 0.0001 |
| LOC100166877 | UDP-glucose 6-dehydrogenase | -0.59 | 0.0002 |
| LOC100162429 | dihydrolipoyl dehydrogenase, mitochondrial | -0.37 | 0.0013 |
| LOC100159914 | cuticular protein-like precursor | -5.40 | 0.0017 |
| LOC100574903 | histone-lysine N-methyltransferase eggless | -0.44 | 0.0018 |
| LOC100570344 | esterase E4 | -1.70 | 0.0045 |
| LOC100574272 | actin-1, partial | -1.25 | 0.0054 |
| LOC100574964 | juvenile hormone acid O-methyltransferase isoform X2 | -0.40 | 0.0059 |
| LOC100574964 | bifunctional lycopene cyclase/phytoene synthase | -1.19 | 0.0060 |
| LOC100166213 | succinate dehydrogenase | -0.32 | 0.0111 |
| LOC100167046 | probable phosphoglycerate kinase | -0.32 | 0.0138 |
| LOC100164251 | pyruvate dehydrogenase E1 component subunit beta, mitochondrial | -0.32 | 0.0157 |
| LOC100162792 | isocitrate dehydrogenase | -0.35 | 0.0226 |
| LOC100159778 | glycogen phosphorylase isoform X1 | -0.31 | 0.0324 |
| Y-y | yellow-y precursor | -0.74 | 0.0372 |
| LOC100159795 | protein phosphatase 1 regulatory subunit 3B isoform X1 | -0.50 | 0.0421 |
| cprr1-4 | RR1 cuticle protein 4 precursor | -0.42 | 0.0507 |
| LOC100163179 | protein takeout isoform X2 | -0.82 | 0.0521 |


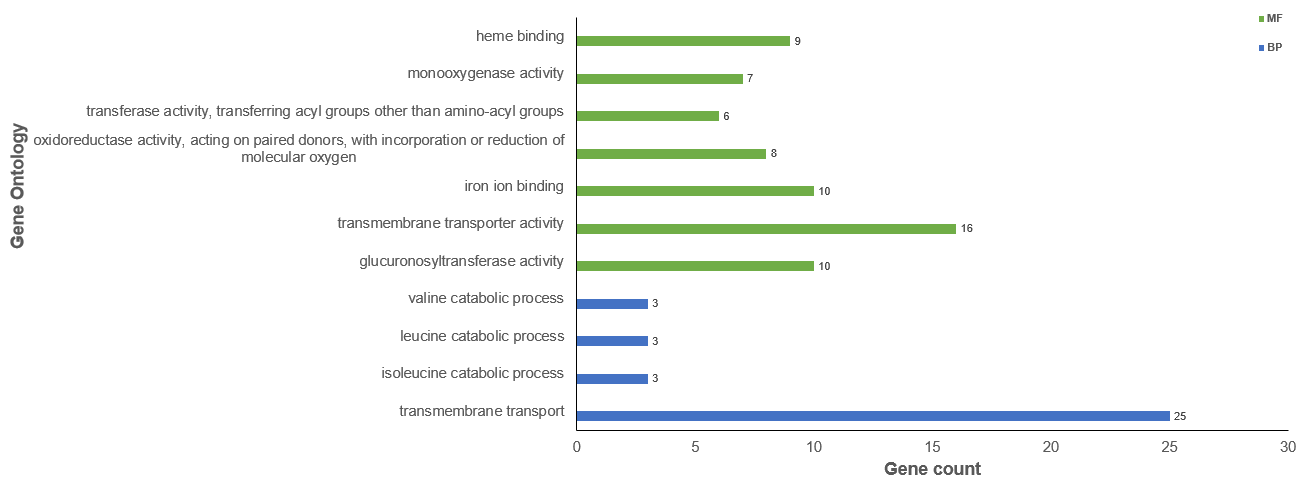


**Figure 4. Gene Ontology analysis for N127 pale vs N127 red.** The colours of the bars represent the intensity of the different GO term. Values represent the number of genes count enriched for each term.


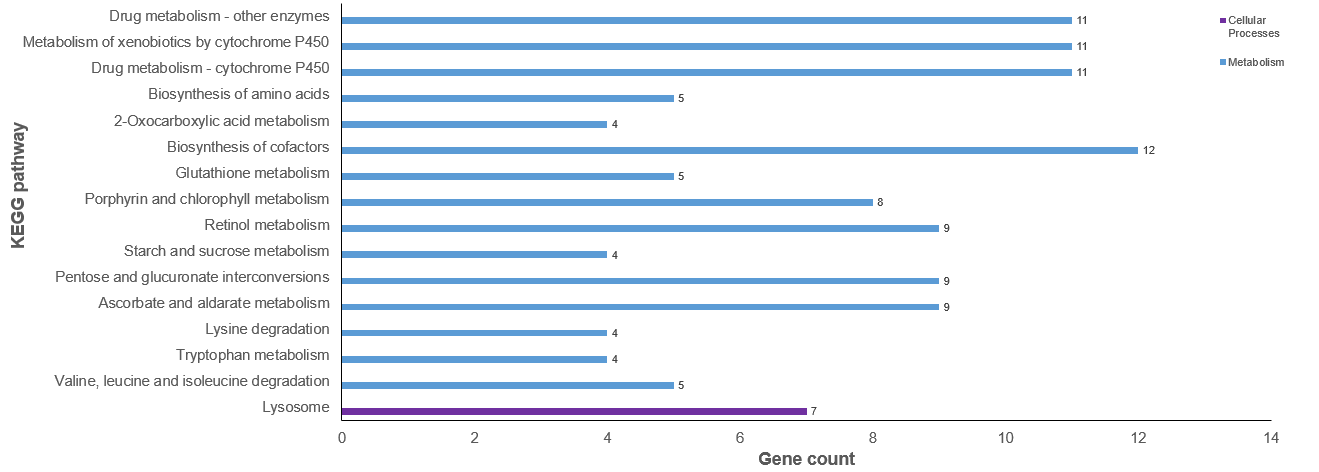


**Figure 5. KEGG analysis for N127 pale vs N127 red.** The colours of the bars represent the KEGG pathway enrichment, and the values represents the number of genes that were enriched for the kegg pathway.

**Table 4. A subset of differentially expressed genes between N127 red and N116 wingless aphids from the full list that may be important in regulating the differences in stress response.**

| Gene ID | Protein Information from NCBI | log2fold | p-value adjusted |
| --- | --- | --- | --- |
| gene-LOC100570971 | protoheme IX farnesyltransferase, mitochondrial-like, partial | 6.60 | 7.86E-32 |
| gene-LOC100159685 | takeout-like precursor | 6.48 | 4.87E-25 |
| gene-tor | carotene dehydrogenase | 7.34 | 6.16E-24 |
| gene-LOC100165740 | retinol dehydrogenase 13 | 4.08 | 4.08E-19 |
| gene-LOC100168000 | probable G-protein coupled receptor Mth-like 2 | 3.39 | 1.63E-18 |
| gene-LOC100168775 | histone deacetylase 8 isoform X1 | 1.70 | 1.69E-16 |
| gene-LOC100168479 | esterase E4 | 0.83 | 1.08E-15 |
| gene-LOC100574469 | heat shock 70 kDa protein cognate 4 | 0.88 | 5.59E-14 |
| gene-LOC100168987 | UDP-glucuronosyltransferase 2B13 isoform X1 | 8.5 | 1.13E-20 |
| gene-LOC100568695 | G-protein coupled receptor Mth2 | 2.05 | 5.60E-11 |
| gene-LOC100163097 | histone deacetylase complex subunit SAP18 | 0.52 | 6.10E-10 |
| gene-LOC100162836 | cytochrome P450 4C1-like isoform X1 | 2.46 | 3.91E-06 |
| gene-cp60 | cuticular protein 60 precursor | 1.70 | 4.03E-06 |
| gene-LOC100158748 | histone acetyltransferase KAT7-like | 3.48 | 9.23E-05 |
| gene-LOC100166213 | succinate dehydrogenase | 0.56 | 0.000221692 |
| gene-LOC100159282 | cuticle protein 7 | 1.29 | 0.00038551 |
| gene-LOC100162620 | leucine-rich repeat extensin-like protein 2 | 1.63 | 0.001368365 |
| gene-LOC100569254 | juvenile hormone acid O-methyltransferase isoform X2 | 0.44 | 0.002220169 |
| gene-LOC100167954 | glyceraldehyde-3-phosphate dehydrogenase-like | 4.46 | 0.022191049 |
| gene-LOC100168026 | heat shock protein 70 B2 | 0.47 | 0.025121779 |
| gene-ORF3 | chemosensory protein-like precursor | 1.20 | 0.000444226 |
| gene-LOC100158682 | set1/Ash2 histone methyltransferase complex subunit ASH2 | 0.38 | 0.003814381 |
| gene-LOC100161104 | bifunctional lycopene cyclase/phytoene synthase | -2.03 | 1.32E-27 |
| gene-LOC100160300 | juvenile hormone epoxide hydrolase 1 | -2.19 | 3.40E-24 |
| gene-LOC100169464 | insulin-like peptide receptor isoform X1 | -0.90 | 5.78E-14 |
| gene-LOC100169645 | heat shock protein 75 kDa, mitochondrial | -0.61 | 6.75E-12 |
| gene-LOC100572714 | V-type proton ATPase subunit B | -0.98 | 3.37E-09 |
| gene-LOC100161380 | phytoene desaturase | -1.14 | 1.95E-07 |
| gene-LOC100167145 | heat shock protein 68-like | -3.3 | 8.52E-06 |
| gene-LOC100161053 | ecdysone-induced protein 78C isoform X3 | -0.73 | 4.35E-05 |
| gene-LOC100161475 | fructose-1,6-bisphosphatase 1 | -1.27 | 7.62E-05 |
| gene-LOC100162796 | homeotic protein spalt-major isoform X2 | -0.68 | 0.000105599 |
| gene-LOC100161043 | trehalase isoform X2 | -0.82 | 0.000231659 |
| gene-Idgf | imaginal disk growth factor precursor | -0.75 | 0.000583239 |
| gene-LOC100168563 | heat shock protein 60A isoform X2 | -0.36 | 0.00091144 |
| gene-LOC100160060 | protein gustavus isoform X4 | -0.30 | 0.000985634 |
| gene-LOC100166128 | fructose-bisphosphate aldolase | -0.44 | 0.001579133 |
| gene-LOC100161594 | short-chain specific acyl-CoA dehydrogenase, mitochondrial | -0.85 | 0.00480125 |
| gene-LOC100159332 | bifunctional lycopene cyclase/phytoene synthase | -1.41 | 0.007712379 |
| gene-LOC100159694 | DNA N6-methyl adenine demethylase | -0.46 | 0.008880593 |
| gene-Ecr | ecdysone receptor isoform A | -0.30 | 0.013408222 |
| gene-LOC100168097 | forkhead box protein O | -0.45 | 0.01377634 |
| gene-Awd1 | abnormal wing discs 1 isoform X1 | -0.31 | 0.015639336 |
| gene-LOC100159050 | phytoene desaturase | -0.76 | 0.015755308 |
| gene-LOC100574964 | bifunctional lycopene cyclase/phytoene synthase | -0.87 | 0.026981725 |
| gene-LOC100167980 | homeobox protein homothorax isoform X2 | -0.37 | 0.031670486 |
| gene-cprr1-2 | RR1 cuticle protein 2 precursor | -3.10 | 8.23E-24 |
| gene-LOC100164834 | Krueppel homolog 1 isoform X3 | -0.73 | 0.000751057 |
| gene-LOC100165557 | trithorax group protein osa isoform X4 | -0.45 | 0.001208728 |
| gene-LOC100160060 | protein gustavus isoform X4 | -0.304 | 0.000985634 |


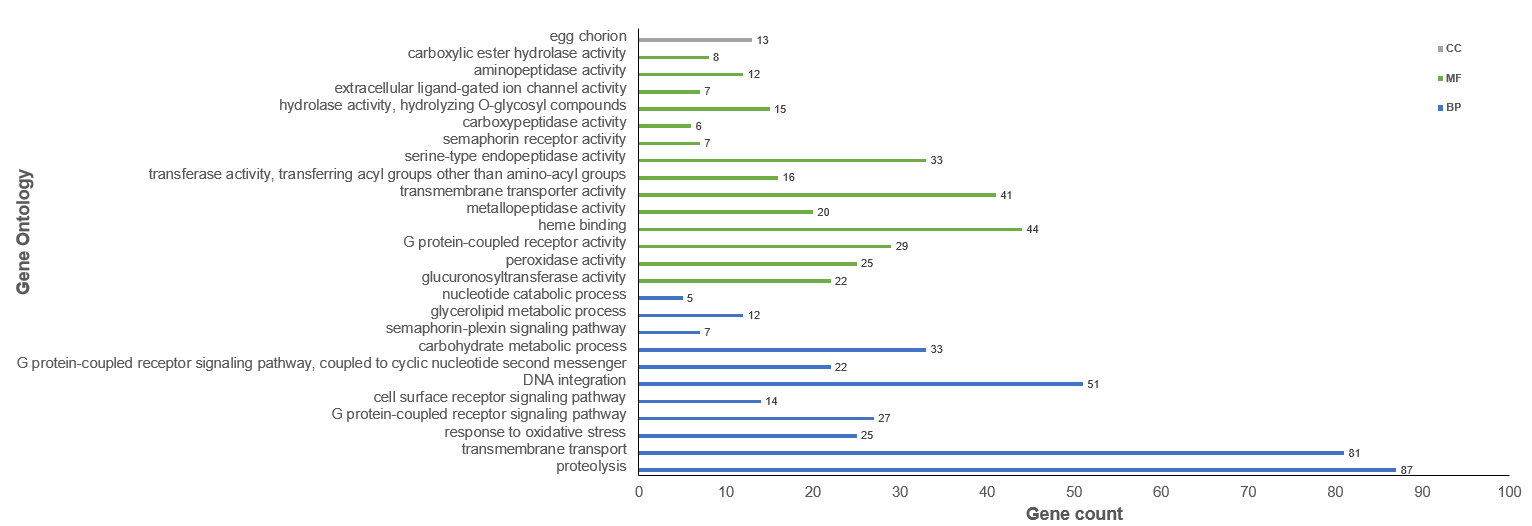


**Figure 6. Gene Ontology analysis for N127 red vs N116 wingless.** The colours of the bars represent the intensity of the different GO term. Values represent the number of genes count enriched for each term.


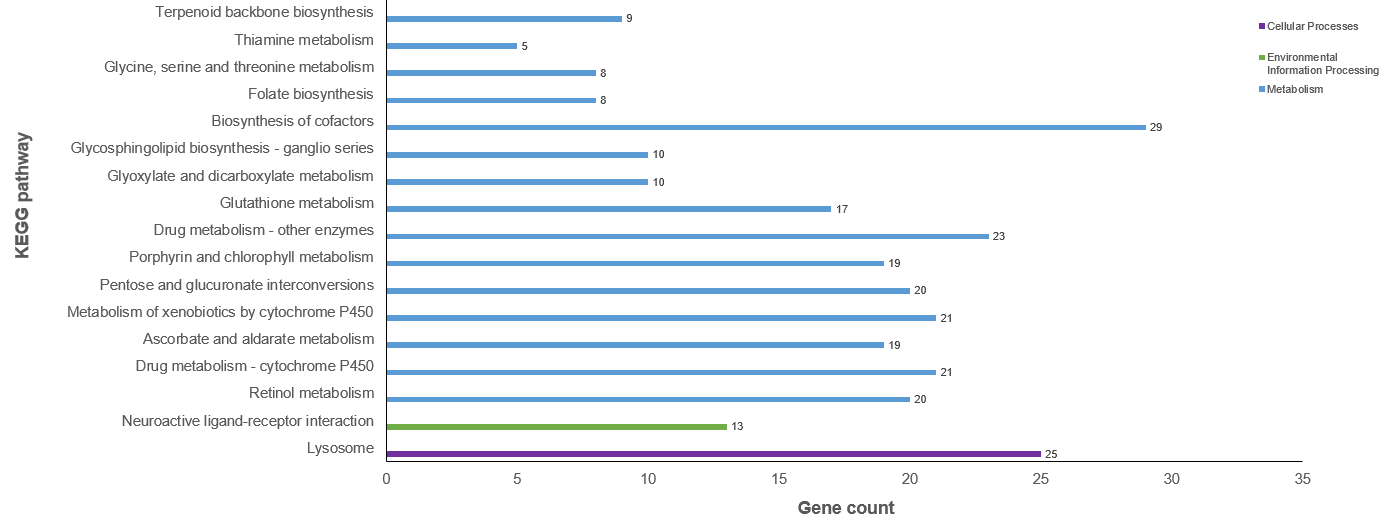


**Figure 7. KEGG analysis for N127 red vs N127 wingless.** The colours of the bars represent the KEGG pathway enrichment, and the values represents the number of genes that were enriched for the KEGG pathway.


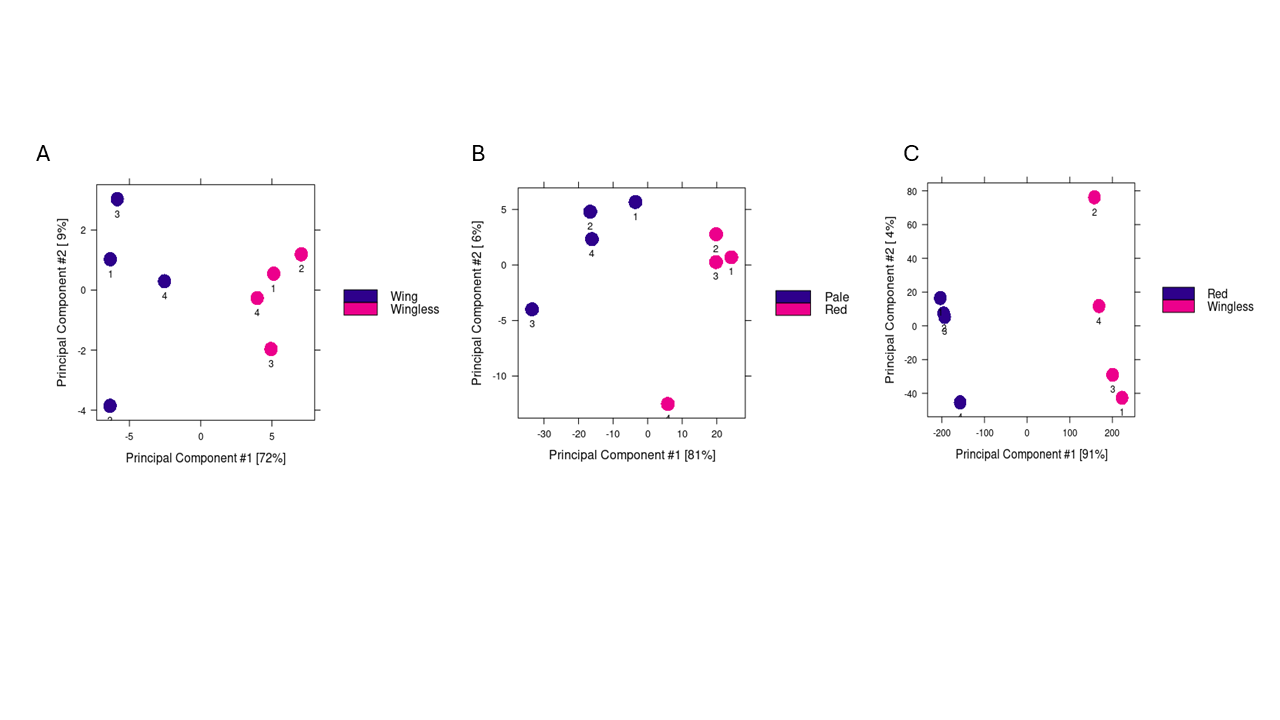


**Figure 8. PCA plot of methylome profiles between pea aphid morphs and genotypes**. A) N116 winged vs N116 wingless. Principal component analysis (PCA) shows clustering of MBD-seq samples by morphs. PC1, explaining 72% of the total variance, separates the N116 winged morphs from N116 wingless (wild type). PC2, explaining 9% of the total variance, differentiates N116 winged from N116 wingless. B) N127 pale vs N127 red. PC1 explains 81% of the total variance and separates the N127 pale morphs from N127 red (wild type),while PC2, explaining 6% of the total variance, captures variation such as individual differences within morphs, or environmental effects between N127 pale vs N127 red. C) Full PCA plot with wild type and morphs, the two aphid genotype cluster into two groups.

**Table 5. Number of reads for each sample for MBD-seq.**

| Strain | sample | Number of input reads | | Uniquelymappedreadsnumber |
| --- | --- | --- | --- | --- |
| 116_winged | SY1 | 35,934,737 | 26,912,328 | |
| 116_winged | SY2 | 51,949,581 | 36,421,975 | |
| 116_winged | SY3 | 33,886,998 | 25,753,730 | |
| 116_winged | SY4 | 47,104,876 | 34,785,149 | |
| 116_wingless | SY5 | 71,022,066 | 48,383,607 | |
| 116_wingless | SY6 | 25,975,306 | 17,331,623 | |
| 116_wingless | SY7 | 55,675,613 | 35,070,243 | |
| 116_wingless | SY8 | 42,200,252 | 26,175,559 | |
| 127_pale | SY9 | 136,314,611 | 82,044,190 | |
| 127_pale | SY10 | 119,965,881 | 73,426,975 | |
| 127_pale | SY11 | 64,967,752 | 43,760,420 | |
| 127_pale | SY12 | 72,992,090 | 48,246,452 | |
| 127_red | SY13 | 64,817,635 | 37,870,028 | |
| 127_red | SY14 | 144,120,572 | 91,342,916 | |
| 127_red | SY15 | 97,564,295 | 62,021,661 | |
| 127_red | SY16 | 58,242,173 | 34,805,227 | |

**Table 6. All four most hypermethylated and the top 10 hypomethylated in N116 winged and N116 wingless aphi****ds.**

| Gene | Chromosome | Fold-change |
| --- | --- | --- |
| Uncharacterized | X | 0.48 |
| disks large homolog 1 | X | 0.62 |
| myocardin-related transcription factor A | X | 0.55 |
| nucleotide exchange factor SIL1 | X | 0.38 |
| Uncharacterized | X | -0.47 |
| sentrin-specific protease 1-like | X | -0.47 |
| Uncharacterized | X | -0.45 |
| kelch-like protein 2 | X | -0.45 |
| E3-independent) E2 ubiquitin-conjugating enzyme UBE2O-like | X | -0.45 |
| peroxisomal membrane protein PEX13-like | X | -0.44 |
| Uncharacterized | X | -0.44 |
| eukaryotic translation initiation factor 4 gamma 3-like | X | -0.43 |
| Uncharacterized | X | -0.43 |
| 26S proteasome regulatory subunit 4 | X | -0.42 |

**Table 7. Top ten most hypermethylated and hypomethylated genes in N127 pale and N127 red aphids.**

| Gene | Chromosome | Fold-change |
| --- | --- | --- |
| soluble guanylate cyclase 88E | A3 | 1.95 |
| UPF0454 protein C12orf49 homolog | A1 | 1.44 |
| translocase of outer mitochondrial membrane 20 | A1 | 0.91 |
| V-type proton ATPase 116 kDa subunit a | A1 | 0.91 |
| Uncharacterized | A2 | 0.79 |
| mitogen-activated protein kinase 13-A | A1 | 0.79 |
| collagen alpha-1(XI) chain | A1 | 0.69 |
| Uncharacterized | X | 0.63 |
| rad23 protein | A1 | 0.62 |
| Histone H2AV | A3 | 0.62 |
| zinc finger protein OZF-like | A2 | -1.25 |
| uncharacterized | X | -1.19 |
| mitochondrial ribosomal protein S18A | X | -1.00 |
| dystonin | A2 | -0.96 |
| f-box only protein 9-like | A2 | -0.89 |
| adapter molecule Crk | X | -0.67 |
| tyrosine-protein phosphatase Lar | A2 | -0.66 |
| Uncharacterized | X | -0.61 |
| Uncharacterized | Unplaced scaffold | -0.60 |
| coiled-coil domain containing 44 | A2 | -0.56 |

**Table 8. Top ten most hypermethylated and hypomethylated in N127 red and N116 red aphids.**

| Gene | Chromosome | Fold-change |
| --- | --- | --- |
| uncharacterized | A3 | 11.92342203 |
| uncharacterized | A1 | 11.22358125 |
| uncharacterized | A1 | 11.15413358 |
| uncharacterized | A1 | 10.92223207 |
| kelch-like protein 2 | A2 | 10.85397283 |
| uncharacterized | A1 | 10.41423335 |
| peroxidase | A1 | 10.40878149 |
| uncharacterized | X | 10.34192549 |
| protein unc-119 homolog B | A1 | 10.32422381 |
| uncharacterized | A3 | 9.801437044 |
| uncharacterized | A2 | -12.10154362 |
| kelch-like protein 3 | X | -11.27950406 |
| kelch-like protein 3 | X | -11.17539917 |
| uncharacterized | A2 | -10.96437326 |
| translation initiation factor IF-2, mitochondrial | A2 | -10.90505053 |
| uncharacterized | X | -10.68701847 |
| AP-1 complex subunit gamma-1 | A2 | -10.54846092 |
| putative nuclease HARBI1 | X | -10.50980174 |
| enoyl-CoA hydratase domain-containing protein 3, mitochondrial | Unplaced scaffold | -10.47703576 |
| protein angel | A2 | -10.38718315 |


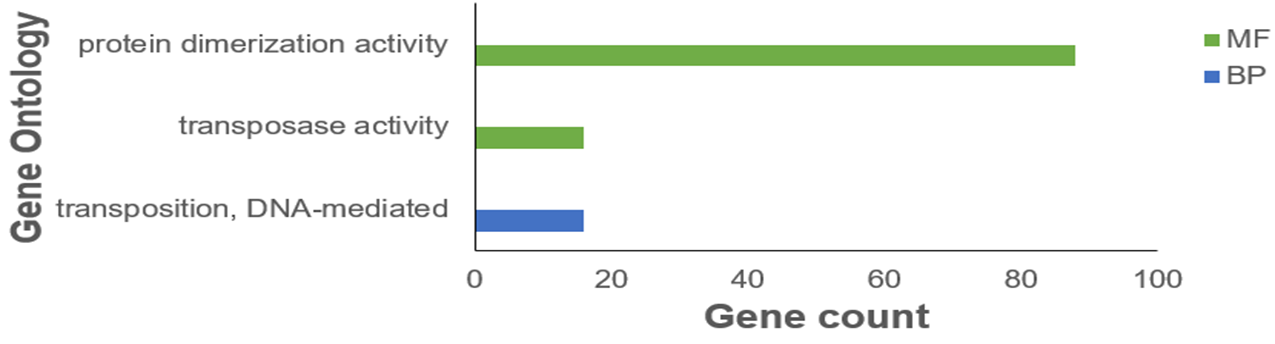


**Figure 9. GO enrichment analysis between N127 red and N116 green wingless aphids for methylation profile**. The colours of the bars represent the the main category in GO term with Y-axis representing the sub category. Values represent the number of genes count enriched for each term.
